# 

**Published:** 1885-05

**Authors:** 


### AMERICAN JOURNAL

—OF—

### DENTAL SCIENCE,

—EDITED BY—

### F. J. S. GORGAS, M. D., D. D. S.

---

VOLUME XIX—THIRD SERIES.

---

1885-86.

SURGEON GEN'L'S OFFICE LIBRARY 105797

BALTIMORE:

SNOWDEN & COWMAN,

LONDON:

TRUBNER & CO., 60 PATERNOSTER ROW.

1886.

### Index to Volume XIX.---Third Series.

A Case in Practice, - - - - - - 33
A Circular, - - - - - - 44
A Peculiar Case, - - - - - - 335
A German Dental Trial, - - - - - - 223
A Series of Questions Pertaining to the Curriculum of the Dental Student, - - - - - 333
A Common Sense Pivot Tooth, - - - 183
A Factor in Tooth Preservation, - - - 305
A Narrow Escape, - - - - - 144
Address by Dr. E. N. Harris, - - - 193
Alcohol, Properties of, - - - - - - 337
Alcoholic Paralysis, - - - - - - 286
Allport, W. W., M. D., D. D. S., - - - 286
Amalgam Question, - - - - - 360
American Dental Association, 38, 90, 514, 571, 572
American Academy of Dental Science, - - - 329
American Medical Association, Section on Oral and Dental Surgery, - - - - - 145
An Appetite for Mortar, - - - - 93
An Old Bullet, - - - - - - 239
Ancient Teeth, - - - - - - - 237
Annual Commencement of Dental Department, University of Maryland, - - - - - - 470, 521
Abscessed Teeth, Treatment Forty Years Ago, - 425
Atmospheric Anæsthesia, - - - - - 502
Arsenical Treatment of Tooth Pulps, - - - 453
Antiseptics for the Oral Cavity, - - - - 119
Alveolar Pyorrhœa, - - - - - - 218
Adams, Dr. J. C., - - - - - 183
Amend, Emil, D. D. S., - - - - - 187
Barrett, W. C., M. D., D. D. S., - - - 385
Beers, W. George, Dr., - - - - - - 1
Bate, C. Spence, F. R. S., - - - - - 348
Bonwill, W. G. A., M. D., D. D. S., - - - 272
Brophy, Truman W., M. D., D. D. S. - - 260

BIBLIOGRAPHICAL.
A Series of Questions for the Dental Student, 283, 333
Chemical Problems, - - - - - 284
Practical Analytical Chemistry, - - - 283
Quiz Questions, - - - - - 284, 333
Dental Bibliography, - - - - - 334
The Physician's Visiting List, - - - 334
Concerning Prof. Gorgas' Questions for Dental Students 473
Recommendations of Prof. Gorgas' Works, - 473
Tabulæ Anatomical Osteclogiæ, - - 478
Vicks' Floral Guide for 1886, - - - 479
California State Dental Law, - - - 41
Calcification of the Teeth, - - - - 96
Curtis, G. L., D. D. S., - - - - 134
Creosote in Dentition, - - - - - 239
Cocaine, - - - - - - 278, 472
Correspondence, - - - - - - 281
Correction, - - - - - - 382, 472
Concerning Prof. Gorgas' Works, - - - 476
Chicago Dental Society, - - - - 520
Capsicum Bags, - - - - - - 526
Cocaine, Hydrochlorate of, - - - - 472
Cocaine Solutions, Preservation of, - - - 528
Chloride of Zinc as a Disinfectant, - - - 432
Clapp, Dr. Dwight M., - - - - - 274
Crown-Work, - - - - - - 141
Capsicum Plaster, New, - - - - - 143
Celluloid, Effects of Steam on, - - - 25
Children's Teeth, - - - - - - 1
Chemistry in Life ana in Death, - - - 171
Caries and Necrosis of Alveoli of Right Superior Maxilla and Chronic Inflammation of Antrum, due to Excessive Salivary Concretions and a Neglected Pulpless Molar, 548
Chicago College of Dental Surgery, - - - 569
Dental Department, University of Maryland, 136, 280, 521
Dentistry in Foreign Countries, - - - 30
Dangers of Wearing Small Dentures, - - - 92
Dental Hygiene, - - - - - - 134
Death from Inhaling Nitrous Oxide, - - - 138
Discard Oxy-Phosphates, - - - - - 142

Dental Caries, Etiology of, - - - - 318
Dodge, J. Smith, M. D., D. D. S , - - - 299
Diagnosis and Treatment of Cystic Tumors of the Jaws, 265
Dotterer, Louis P., D. D. S., - - - 269, 467
Diseases of the Period of Dentition, - - - 385
Disgust for Decayed Teeth, - - - 431
Discoloration of Gold Fillings, - - - - 366
Deep-Seated Abscesses, - - - - - 370
Dental Literature and Dental Education, Report on - 433
Dental Spiritualism, - - - - - - 504
Dentition, Diseases of, - - - - - 385
Dental Section of International Medical Congress, 428, 429
Dead Teeth in the Jaws, - - - - 260
Edwards, H. H., D. D. S., - - - - 33
Etiology of Dental Caries, - - - - 318
Effect on Offspring of Consanguineous Marriages, - 192
Excision vs. Extraction, - - - - - - 348
Exsection of Inferior Dental Nerve for Relief of Paroxysms of Lower Jaw, - - - - - - 467
Extraordinary Effects of Drugs, - - - - 479
Effects of Amalgam Fillings upon the System, - 563
Fletcher, Thomas, - - - - - - 185
Filling Materials, - - - - - - 47
First District Dental Society of New York, - - 406
Forty Years Ago, Treatment of Pulpless and Abscessed Teeth, - - - - - - - 425
Food for Infants, - - - - - - 323
Forbes, Dr. Isaiah, - - - - - - 232
Fracture of Jaw and Treatment, - - - 233
For Indigestion. - - - - - - 239
Galvanism for Neuralgia, - - - - - 191
Gas Furnaces vs. Coal or Coke, - - - 518
Georgia Dental Law, - - - - - - 326
Georgia State Dental Society, - - - - 289
Glycerine in Dryness of the Tongue, - - - 528
German Trial against Dentists using the title, D. D. S, 223, 568
Gold Fillings, Discoloration of, - - - - 366
Gaston, George C., - - - - - - 223
Hardman, J., D. D. S., - - - - - 360
Harris, Edward N., D. D. S., - - - - - 193

Hayes, Fred. Hooper, - - - - 323
Holmes, Dr. Oliver Wendell, Toast of, - - - 471
Headache, - - - - - - 90
Hutchinson, Mr. Jonathan, - - - - 576
Hydrochlorate of Cocaine, - - - - 422
International Medical Congress, 235, 330, 428, 429, 468, 524
Indigestion, - - - - - - 239
Incipient Stages of Inebriety, - - - 95
Illinois State Dental Society, - - - - 521
Ives, Chas. F., D. D. S., - - - - 354
Kingsbury, Chas. A., D. D. S., - - - - 30
Kulp, Dr., - - - - - - - 491
Land, H. C., D. D. S., - - - - - 518
Maryland State Dental Association, - - 374
Marshall, John S., D. D. S., M. D., - - - 370
Mend a Broken File, - - - - 527
Morseman, A., M. D., D. D. S., - - - 453
Mills, Geo. A., D. D. S., - - - - 218
Mortality of Infants, - - - - - 94
Miller, W. B., D. D. S., - - - - - 119
Morrison, W. N., D. D. S., - - - - 180
National Association of Dental Examiners, - - 228
National Association of College Faculties, - - 230
New Experiments and Observations on Hydrobromic
Ether or the Bromide of Ethyl as an Anæsthetic, - 529
Nerve Centres and the Teeth, - - - - 299
Nervous Energy, - - - - - 241
Non-Fistulous Abscess, - - - - 491
Nourishing the Tissues of the Teeth, - - - 288
Odonto-Chirurgical Society, - - - 480
Odontological Society of Great Britain, - 14, 126, 493
Ottofy, Louis, D. D. S., - - - - 504
Opposing Baltimore Medical Colleges—Correction, - 472
Oxy-Phosphate, - - - - - 336
OBITUARY.
Dr. Isaiah Forbes, - - - - - 232
Dr. John P. Holmes, - - - - - 383
Dr. John M. Riggs, - - - - 383
On the Availibility of Certain Antiseptics in the
Prophylactic Treatment of the Oral Cavity, - 119

Palmer, S. B., D. D. S., - - - - - - 366
Preservation of Cocaine Solutions, - - - - 528
Parsons, E., D. D. S. - - - - - - 241
Peirce. C. N., D. D. S., - - - - - 305
Pulpless Teeth, - - - - - - - 253
Peroxide of Hydrogen, - - - - - 285
Pivot Tooth, A Common Sense, - - - - 183
Poisonous Effects of Amalgam Fillings, - - - 185
Practice of Dentistry in Germany, - - - - 187
Prevention of Bad Teeth, - - - - - 573
Prosecution of a Dentist, - - - - - 574
Rambo, Samuel, M. D., D. D. S., - - - - 425
Report on Dental Literature and Education, - - 433
Riggs' Disease, - - - - - - 218
Right and Left-Handedness, - - - - - 464
Richards, W. H., D. D. S., - - - - - 502
Reflex Pain, - - - - - - - 240
Rubber, Effects of Steam on, - - - - 25
Regulating Teeth, - - - - - - 45
Section of Oral and Dental Surgery in the International
Medical Congress, - - - - - 375
Separating Teeth, Some Methods of, - - - 274
Some of the Physiological and Therapeutical Properties
of Alcohol, - - - - - - - 337
Smith, John S., D. D. S., - - - - - 265
Seabury, F. W., D. D. S., - - - - - 25
Stevens, J. P., M. D. - - - - - - 337
Sore Mouth Under Plates, - - - - - 384
Saving Roots. - - - - - - - 236
Southern Dental Association, - - - - 49, 97
Some Modifications of the Antiseptic Method, - - 139
Steam and its Effects on Rubber and Celluloid, - 25
Shaw, Parsons, D. D. S., - - - - - - 164
Thackston, W. W. H., M. D., D. D. S., - - - 433
To Mend a Broken File, - - - - - 527
Treatment of Deep-Seated Abscesses without External
Incisions, - - - - - - 370
Thoroughness, - - - - - - - 269
The Most Popular Medicines, - - - - 238
Teeth without Plates, - - - - - - 95

Teeth, Calcification of, - - - - - 96
The Reason why the Dental Department of the University of Maryland deems it Inexpedient to Join the National Association of College Faculties, - - - 136
Teeth in Different People, - - - - - 164
The Reckless Sacrifice of Tooth Substance in Devitalized Teeth, - - - - - - 180
The Wisdom Teeth, - - - - 554
To State and Local Dental Societies, - - - 570
The Maryland Dental Law Amendments, - - 571
The Fluctuations in Cocaine, - - - - 575
Teague's, Dr. B. H., Depressed Engine Disks, - 572
University of Maryland, Dental Department, - - 280
University of California, - - - - 469
Virginia State Dental Law, - - - - 566
Virginia State Dental Association, - - - 328
Watt, George, M. D., D. D. S., - - 171, 422
White, Richard, L. D. S., - - - - 328
Wilson, Dr. - - - - - - 253
What Fillings Should We Use, - - - 272

### AMERICAN DENTAL ASSOCIATION.

The twenty-fifth annual meeting of the American Dental Association will be held at Minneapolis commencing August 4th. 1885.

The present prospects are that the meeting will be an unusually large one.

The railroad rates have been secured at an unprecedentedly low rate. Tickets for the round trip from New York to Minneapolis and return will be furnished for $24.00. Round trip from New York to Chicago and return $18.00. Round trip from Chicago to Minneapolis and return $6.00,

At present it will be necessary for those wishing these tickets to secure them in Chicago. Later we may be able to make arrangements by which they can be secured at different points east. By sending check for tickets to chairman of committee of arrangements the tickets will be promptly forwarded.

Negotiations are pending for rates from other points that the committee anticipate will accommodate all, and more definate information will be given in later journals and also in a circular sent to every member.

The hotel rates are as follows:

| | | |
|---|---|---|
| West Hotel, | - - | $4.00 per day. |
| Nicollet House, | - | $3.00 " " |
| National Hotel, | - - | $2.00 " " |

It is expected that a reduction from these will made. It is hoped that members having any new facts or ideas in regard to theory or practice will come prepared to present them in connection with the section work. Any one having anything new in the way of appliances will be given an opportunity to demonstrate their use during the half day that will be devoted to clinics.

ATTRACTIONS AND EXCURSIONS.

Come equipped with guns and fishing tackle. While the interest and benefit of the meetings, the attractions of the trip and the beautiful city where we meet are too well known to need special mention it may not occur to all that they will find themselves in Minnesota in one of the finest of hunting and fishing countries. Minnesota is especially famous for its prairie chicken and grouse shooting, and its fine fishing grounds.

It is estimated that there are no less than 10,000 lakes dotting the state.

If one wishes a still greater variety of scenery, to see a new wild and picturesque country, to draw out the big brook trout, the black bass, the "gamy" pickerel and the mighty muskallonge from the cold waters of the Lake Superior region, in fact to enjoy the finest fresh water fishing in the world, a round trip ticket from Chicago to Ashland and return will be furnished them for $10.00.

A STILL GREATER ATTRACTION.

(If one more were needed) is offered in shape of a ten day's excursion to the far famed "Yellow Stone National Park," immediately upon close of the association, provided a sufficient number send in their names to warrant the securing of special cars and special rates. The committee believe that when so far on the way as Minneopolis many will wish to avail themselves of this opportunity of seeing the grandest scenery in the world. The entire expense for round trip from Minneapolis including rail transportation, Pullman Sleeping Car Fares, meals on Northern Pacific Dining Cars, hotel accommodations, five days in the Park and stage transportation, will be $120.00. A circular describing the magnificent scenery in full will be sent to every member of the American Dental Association at an early day. Others than members who may contemplate going will receive the same by making application for it.

Come one, come all and bring your wives along. It will be a trip that ladies will especially enjoy. Those wishing to go to Yellow Stone Park will please send in their names at an early day that all arrangements may be speedily and satisfactorily completed.

For further information address J. N. Crouse, 2101 Michigan Avenue. Chairman of Committee of Arrangements.

### To Readers and Correspondents.

Communications intended for publication, and exchange journals and papers should be addressed to the EDITOR OF THE AMERICAN JOURNAL OF DENTAL SCIENCE, 259 N. Eutaw St., Baltimore, Md. All letters relating to the business of the JOURNAL, or containing cash remittances, to be directed to Messrs. SNOWDEN & COWMAN. Articles for publication should be received by the editor at least three weeks previous to the first month on which the number in which it is designed for them to appear, is issued.

### CONTENTS:

ART. I—The Teeth of Children. By Dr. W. George Beers ..... 1

ART. II—The Odontological Society of Great Britain ..... 14

ART. III—Steam, and its Effects on Rubber and Celluloid when Properly Applied. By Dr. F. W. Seabury ..... 25

ART. IV—Dentistry in Foreign Countries. By Dr. Charles A. Kingsbury ..... 30

ART. V—A Case in Practice. By H. H. Edwards D D. S., ..... 33

American Dental Association ..... 38

### EDITORIAL, ETC.

California State Dental Law ..... 41

A Circular ..... 44

### MONTHLY SUMMARY.

Regulating Teeth ..... 45

Filling Materials ..... 47

☞ The AMERICAN JOURNAL OF DENTAL SCIENCE will contain fifty pages of reading matter in each number, making a volume of about *Six Hundred* pages,

EXCLUSIVE OF ADVERTISEMENTS.

